# Analysis of non-conjugated steroids in water using paper spray mass spectrometry

**DOI:** 10.1038/s41598-020-67484-7

**Published:** 2020-07-01

**Authors:** Fred. P. M. Jjunju, Deidre E. Damon, David Romero-Perez, Iain S. Young, Ryan J. Ward, Alan Marshall, Simon Maher, Abraham K. Badu-Tawiah

**Affiliations:** 1 Department of Electrical Engineering and Electronics, University of Liverpool, Brownlow Hill, Liverpool, L69 3GJ UK; 2 Department of Chemistry and Biochemistry, The Ohio State University, Columbus, OH 43210 USA; 3 Institute of Integrative Biology, Biosciences Building, University of Liverpool, Liverpool, L69 7ZB UK

**Keywords:** Analytical chemistry, Mass spectrometry, Analytical biochemistry

## Abstract

A novel strategy for the direct analysis of non-conjugated steroids in water using paper spray mass spectrometry (PS-MS) has been developed. PS-MS was used in the identification and quantification of non-conjugated (free) steroids in fish tank water samples. Data shown herein indicates that individual amounts of free steroids can be detected *in aqua* as low as; 0.17 ng/µL, 0.039 ng/µL, 0.43 ng/µL, 0.0076 ng/µL for aldosterone, corticosterone, cortisol, and *β*-estrone, respectively, and with an average relative standard deviation of ca. < 10% in the positive ion mode using PS-MS/MS. Direct detection of free steroids in a raw water mixture, from aquaculture, without prior sample preparation is demonstrated. The presence of free steroids released in fish water samples was confirmed via tandem mass spectrometry using collision-induced dissociation. This approach shows promise for rapid and direct water quality monitoring to provide a holistic assessment of non-conjugated steroids *in aqua*.

## Introduction

Steroids are a class of biologically active compounds with 17 carbon atoms arranged in a four-member ring system that are vital in a wide range of biological functions (i.e. cellular signaling, growth, development etc.)^1^. The main steroids of biological importance include cholesterol, sterols, bile acids, corticosteroids (e.g., cortisol, corticosterone) and sex steroids (e.g., estrogens, androgens)^2^. There is a growing interest in the development of new analytical tools for steroid analysis due to the potential of steroids as biomarkers for a variety of diseases, their potential misuse to enhance performance in sports^3–5^, and their use in animal production and to monitor animal welfare (e.g., cortisol, the primary stress hormone)^6^.

Aquaculture for food production has been valued globally at around $160 billion^7^. High stress (in fish) is reported during transport, capture and stocking procedures, which can be correlated with plasma cortisol levels^8–10^. This leads to increased energy consumption, decreased feeding, increased susceptibility to disease and/or impaired survival^11,12^. Due to the increasing importance of aquaculture as a means of food production and the impact of stress on production efficiency, fish welfare is becoming an increasingly important issue for the industry. Therefore, there is a pressing need for new analytical innovations to monitor fish health and welfare. In this study we focus on establishing direct mass spectrometric approaches for rapid steroid measurements, including cortisol, which has implications for measuring stress in fish *in aqua* as an early warning of welfare issues^13^, as well as for environmental monitoring^14^, without causing undue stress or destruction of the animals.

The analysis and quantification of steroids is important due to their vital biological roles (i.e. development, reproduction regulations) in animals^15^. For example, *β*-estrone, a sex hormone, possesses potent antioxidant properties and plays important roles in maintaining normal reproductive and non-reproductive functions^15^. However, even in trace amounts, *β*-estrone may have adverse effects on humans and the aquatic ecosystem^16^. It is therefore essential to reliably determine trace amounts (at environmentally relevant concentrations) of *β*-estrone and other hormones in water. In relation to monitoring fish, *in aqua* steroid measurements are non-invasive (unlike blood tests) and therefore do not require any physical intervention (e.g., anesthetic injection, handling, bleeding). This ensures that results are not influenced by any stress incurred during sampling, whilst also allowing concurrent monitoring of the animal behavior^17^.

Radioimmunoassay (RIA) is the most common analytical method used to measure steroids with good sensitivity and reproducibility^18,19^. RIA is gradually being replaced by enzyme-linked immunosorbent assays (ELISA) because of legislation relating to laboratory waste^20,21^. However, there are well-known difficulties associated with ELISA: as an immunoassay, it is sensitive to incubation time and temperature making standardization difficult, it is prone to cross reactivity with other analytes in the sample and ELISA can suffer from limited sensitivity^21^. A combination of solid phase extraction (SPE) followed by liquid/gas chromatography (LC/GC) mass spectrometry (SPE/LC/GC–MS) is the principal antibody-free analytical method with little or no hazardous waste accumulated during the detection of steroids in water^22,23^. SPE/LC–MS is a highly sensitive and selective method, which makes it suitable for both qualitative and quantitative steroid analysis^24^. However, SPE/LC–MS is time-consuming and requires relatively extensive sample workup to minimize matrix effects^25,26^. A rapid, high throughput, non-invasive, quantitative methodology for measurement of steroids in water without disturbing the fish, could provide critical information on animals in aquaculture and allow more effective farm management decisions to be made in a timely manner^27,28^.

Ambient ionization mass spectrometry (AI-MS)^29–31^ combined with tandem mass spectrometry (MS/MS) represents a promising solution for the simplification and/or elimination of sample preparation procedures for steroid analysis *in aqua* without impacting on the welfare of the animals. AI-MS is becoming widely accepted for the direct analysis of untreated samples, whereby sampling and ionization is conducted in ambient conditions with no (or minimal) sample workup^29,32^. The fact that no sample preparation or prior extraction steps are needed during analysis means that AI-MS analysis workflows are simple, which ultimately reduces the total MS analysis time (from sample to result)^33^. Some of the more popular ambient ionization methods include desorption electrospray ionization (DESI)^34^, direct analysis in real time (DART)^35^, desorption atmospheric pressure chemical ionization (DAPCI)^36,37^, nano-desorption electrospray ionization (nano-DESI)^38^, low temperature plasma (LTP)^39^ and paper spray (PS)^40,41^, amongst others. These methods have been successfully deployed for the analysis and quantification of a wide range of samples in complex mixtures without any sample pre-treatment^32,42–47^.

PS ionization is a relatively new AI-MS method, which has been successfully applied in the direct analysis and quantification of complex molecules, ranging from small organics to large biological molecules including dried blood under ordinary ambient conditions^32,48–50^. With PS-MS sample collection, separation and ionization are combined in one step. As such, it offers potential advantages, including: high-throughput, less solvent waste, ease of use, and the possibility to interface with a portable mass spectrometer because little/no sample preparation is required. This makes PS an attractive option for non-invasive (i.e., without disturbing fish), rapid and potentially on-site analysis, if it can be applied to the measurement of steroids *in aqua*. For example, recent studies utilizing PS have been successfully applied to the analysis of amines in water samples^51,52^, petroleum oil samples^53^, pesticides^54^, and a wide range of other analytes^55^.

In the present study, we report the detection and quantification of water-borne non-conjugated (free) steroids (i.e. cortisol, corticosterone, aldosterone and *β*-estrone) in crude water samples collected from industrial fish tanks using PS-MS/MS. The samples are representative of the majority of aquaculture applications, as well as other allied scenarios (e.g., research related fish studies), which often require holistic monitoring of entire stock where *in aqua* steroid levels are relatively high due to the high fish densities encountered (e.g., 10^5^ fish in a single tank is common). Sample preparation was reduced to dilution of the standard model compounds in methanol while the raw water samples were analyzed directly as supplied without any pre-treatment or pre-concentration. The ability to identify free steroids *in aqua* can be achieved at trace levels, including *β*-estrone which was detected at part per billion level. The relative standard deviations were < 10% and over two orders of magnitude dynamic range was observed.

## Results and discussion

First, to investigate the potential of PS-MS for steroid analysis and to establish an analytical procedure, the standard model compounds were analyzed and characterized using tandem mass spectrometry followed by quantitative analytical performance measurements. After establishing the analytical approach, 13 raw aquaculture samples were analyzed quantitatively for the four free steroids in question (i.e. cortisol (Mw 362), corticosterone (Mw 346), aldosterone (Mw 360), and *β*-estrone (Mw 270)).

### Direct analysis and solvent optimization of free steroids in the positive ion mode using PS-MS

**Figure 1:**
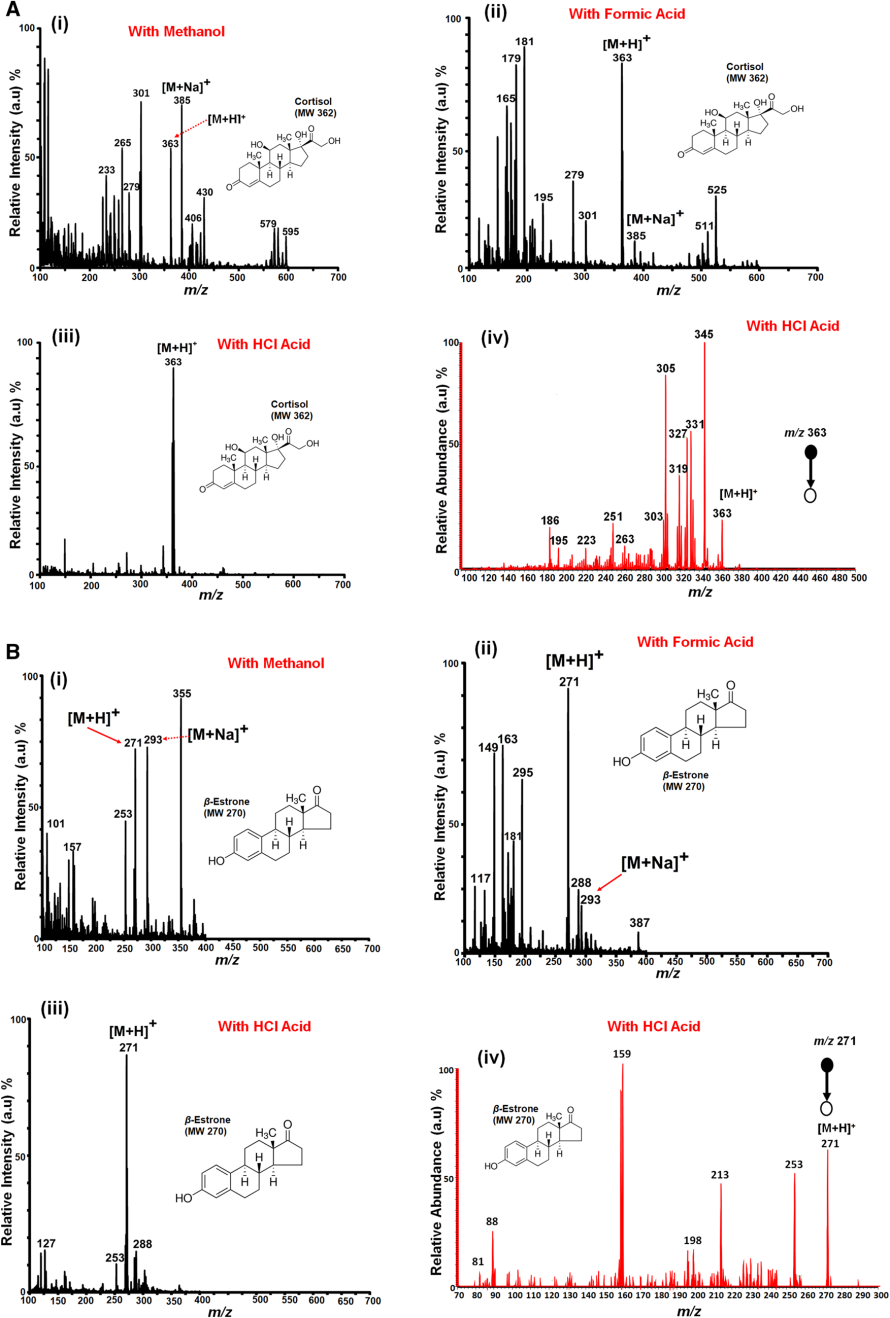
(**A**) Positive ion mode paper spray mass spectra of cortisol (MW 362) model compound analyzed using a bench-top ion trap mass spectrometer for various spray solvents: (**A**) (i) with 20 µL MeOH/H_2_O (1:1), (A) (ii) MeOH/H_2_O (1:1) doped with 500 mM formic acid, (**A**) (iii) MeOH/H_2_O (1:1) doped with 0.05 mM HCl and (A) (iv) shows the MS/MS CID data for the selected protonated molecular ion [M + H]^+^ at *m/z* 363 for the HCl doped spray solvent of (**A**) (iii); (**B**) *β*-estrone (Mw 270), analyzed with/without acid doping in a similar fashion; (**B**) (i) with 20 µL MeOH/H_2_O (1:1), (**B**) (ii) MeOH/H_2_O (1:1) doped with 500 mM formic acid, (**B**) (iii) MeOH/H_2_O (1:1) doped with 0.05 mM HCl and (**B**) (iv) shows the MS/MS CID data for the selected protonated molecular ion [M + H]^+^ at *m/z* 271 for the HCl doped spray solvent of (**B**) (iii). In all cases absolute amounts of analyte were spotted onto a filter paper and ionized in the open air by application of an electric potential (+ 5 kV), 5 µL, viz 5 ng/µL.

Figure 1 shows the recorded PS mass spectra of cortisol (Mw 360) and *β*-estrone (Mw 270) model compounds in positive mode, using [MeOH/H_2_O], 1:1 v/v, as the standard spray solvent. In Fig. 1A (i), an intense sodium adduct ion, [M + Na]^+^, of cortisol at *m/z* 385 and a less intense protonated molecular ion, [M + H]^+^, at *m/z* 363 are observed. The corresponding mass spectrum of *β*-estrone recorded with PS-MS is shown in Fig. 1B (i).

When the standard spray solvent, MeOH/H_2_O, 1:1 v/v, was doped with a weak acid (500 mM of formic acid), an intense protonated molecular ion, [M + H]^+^, at *m/z* 363 for cortisol and *m/z* 271 for *β*-estrone was obtained with the corresponding sodiated peaks diminishing (Fig. 1A (ii), B (ii), respectively). When doping the spray solvent with a stronger acid, 0.05 mM of hydrochloric acid (HCl), the intensity of the protonated peak intensifies and any sodiation is difficult to distinguish from the noise for both cortisol and *β*-estrone (Fig. 1A (iii), B (iii), respectively).

These results are in good agreement with ESI^56^ and MALDI^57^. The effect of adding a dopant such as formic acid (500 mM) or HCl (0.05 mM) to the PS solvent (MeOH/H_2_O, 1:1 v/v) shows that the mechanism of ionization immediately favors the formation of the protonated molecule, [M + H]^+^, by proton transfer. The most likely point of protonation on the steroid molecule is at the hydroxyl (OH) group^58^. Many biologically important molecules, such as the steroids studied herein, cannot be easily ionized using the typical paper spray solvent (methanol/water (1:1, v/v)) due to ion suppression^59^. This is because many biological samples contain salts, and many ions compete for charge^60^. The addition of a dopant such as HCl favors the production of the protonated molecular, [M + H]^+^, ion with little or no fragmentation (Fig. 1A (iii), B (iii)).

Tandem mass spectrometry (MS/MS) with collision induced dissociation (CID) was used to confirm the identity and the chemical structure of the analyzed steroid compounds; i.e., the protonated molecular ion, [M + H]^+^, of the model compounds studied using PS-MS. Figure 1A (iv), B (iv) show the CID mass spectra of cortisol and *β*-estrone obtained for the dissociation of the protonated molecular ion at *m/z* 363 and *m/z* 271, respectively (whereby the spray solvent was doped with HCl). Notice that, upon CID activation (Fig. 1A (iv)), the [M + H]^+^ ion at *m/z* 363 for cortisol resulted in the less intense fragment peak at *m/z* 345 due the loss of water [− 18 Da or − H_2_O] followed by subsequent loss of another water molecule resulting in a higher intense fragment ion at *m/z* 327. A similar CID fragmentation transition was also obtained for *β*-estrone at *m/z* 271 (Fig. 1B (iv)). Water losses due to MS/MS activation are characteristic transitions of most steroids with a hydroxyl group that can be readily lost as a water molecule [− 18 or − H_2_O]. The fragmentation pattern observed confirms the identity and the structure of the model steroid compounds studied. Other model compounds studied include corticosterone (Mw 346) and aldosterone (Mw 360), with full MS and CID data given in the supplementary information (Figures S1 and S2).

Most polar steroids can be readily protonated or deprotonated using PS-MS (in positive or negative ion mode). However, the degree of their protonation is also dependent on their proton affinity (with high proton affinity favoring formation of the protonated molecule)^61^. The addition of dopants can improve the analytical specificity and detection limits for certain analytes in complex mixtures^51^. As observed in Fig. 1, the steroids analyzed, cortisol and *β*-estrone, using PS-MS with an acid dopant (i.e. either formic acid or HCl), yielded dominant protonated molecular ion species, [M + H]^+^, with diminished sodium adducts, [M + Na]^+^, compared to the standard spray solvent MeOH/H_2_O (1:1, v/v). The proton affinities of the steroids studied can be estimated to be in the range 820–870 kJ mol^−1^ based on the method of Prof. Graham Cooks (by using group equivalent effects on the known values of simple analogues)^62^. As such, steroids can be protonated in the presence of proton donating additives such as water, formic acid or HCl (Fig. 1) when analyzed using PS-MS. As demonstrated in this study, the ability to improve the ionization of steroids through protonation ([M + H]^+^) by adjusting the dopant composition is effective. This can be particularly useful when molecular weight confirmation is required in the presence of other competing analyte(s).

**Figure 2:**
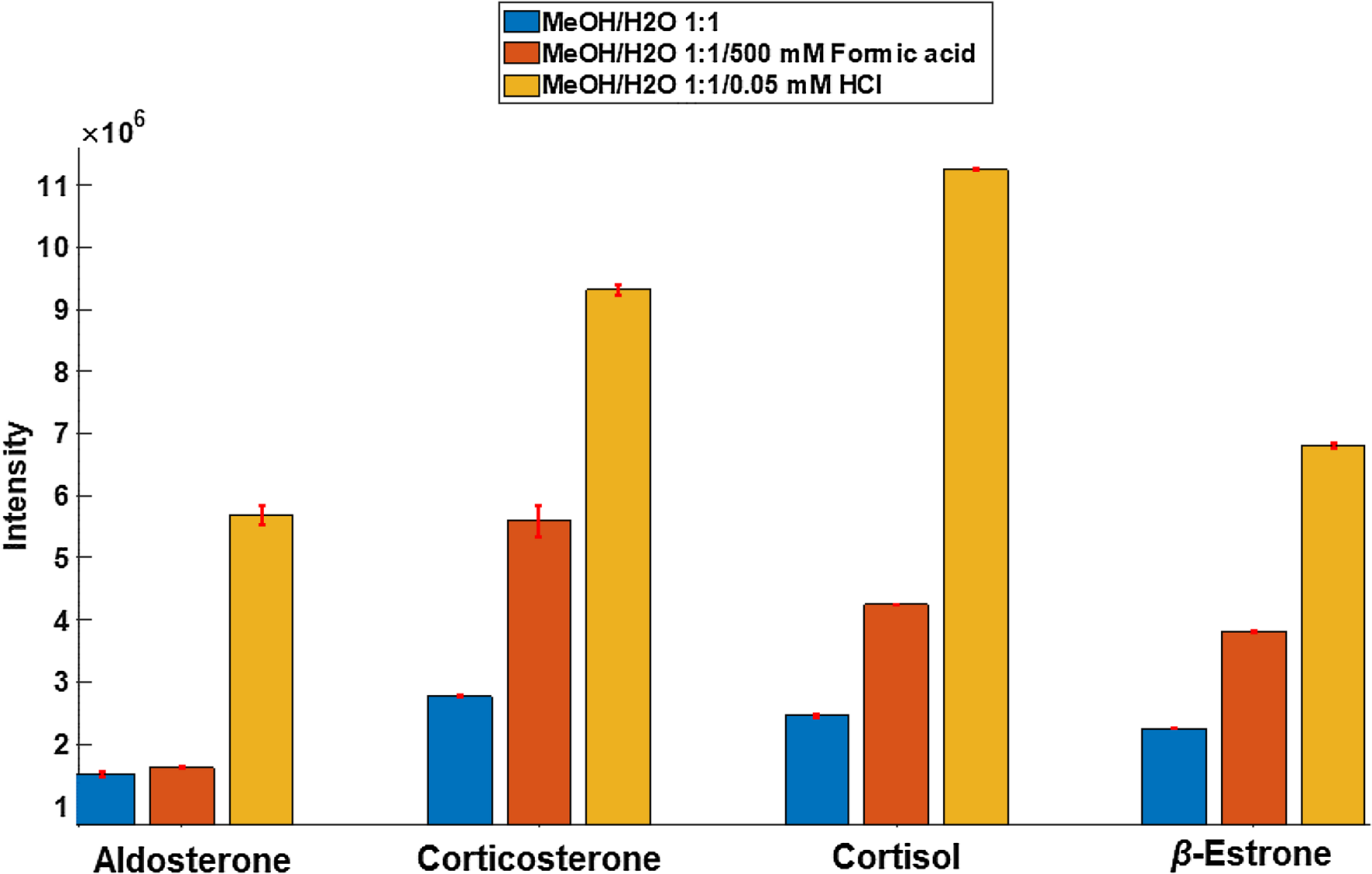
PS MS solvent optimization (MeOH/H_2_O 1:1, v/v, MeOH/H_2_O 1:1/ 500 mM Formic acid, MeOH/H_2_O 1:1/ 0.05 mM HCl) for the analysis of free steroid model compounds (i.e. aldosterone, corticosterone, cortisol and *β*-estrone), whereby each bar represents the mean abundance of the protonated molecular ion peak. Error bars indicate the standard deviation of the samples.

By adjusting the solvent composition, the type of adduct ions obtained can be effectively manipulated in both the positive and negative ion modes. As seen in Fig. 1, this can have a significant bearing on ion formation. The various physical and chemical properties of a solvent (i.e. polarity, volatility, surface tension, etc.) play a significant role in the spray process of PS^63^ and many similarities are observed when compared with conventional electrospray ionization^5^. Typically, the spray solvent composition is adjusted, as well as the sample solvent (where possible), to optimize the spray conditions for the analysis at hand^64,65^. As shown in Fig. 2, the addition of a dopant to the paper spray solvent greatly improves the signal intensity of the target analyte(s) by promoting a favorable ionization mechanism (i.e. protonation [M + H]^+^).

### Quantitative analysis

The free steroid model standards in 5 µL of water dried onto a paper surface and spiked with 5 µg/mL of cortisol-1α,2α-d_2_ were analyzed with MeOH/H2O (1:1, v/v) doped with 0.05 mM HCl. The analytical performance of PS-MS was carried out in the MS/MS mode using CID transitions (*m/z* 361/315, *m/z* 347/293, *m/z* 363/327, *m/z* 271/158; for aldosterone, corticosterone, cortisol, and *β*-estrone, respectively and *m/z* 365/269 for cortisol-1α,2α-d_2_). The intensity ratios of the two transitions were plotted against the known concentrations of the standard to build the calibration curves for the model compounds with a minimum of 5 points over a linear dynamic range of concentrations (0.1–10 µg/mL) covering the expected sample concentration (Figure S3, supplementary information). The limit of detection (LOD) was determined as the concentration that produces a signal more than three times greater than the standard deviation plus the mean value of the blank, in the MS/MS mode using CID. The limit of quantitation (LoQ) was determined as the concentration that results in a signal 10 times greater than the standard deviation plus the mean value of the blank. The LODs of the four model compounds were determined to be 0.17 ng/µL, 0.039 ng/µL, 0.43 ng/µL, 0.0076 ng/µL for aldosterone, corticosterone, cortisol, and *β*-estrone respectively, with a relative standard deviation of ca. < 10%. While the LoQ of these steroids *in aqua* was determined to be 0.39 ng/µL, 0.064 ng/µL, 0.48 ng/µL, and 0.41 ng/µL for aldosterone, corticosterone, cortisol, and *β*-estrone, respectively, in the positive ion mode with HCl (0.05 mM) doped in the standard spray solvent, MeOH/H_2_O (1:1, v/v) (Table 1). These results are in good agreement with previous studies, where chloride ions have been utilized in the selective ionization of carbohydrates using conventional ESI^66–68^.

**Table 1:** Chemical structures and CID product ions of the non-conjugated model standard steroids analyzed.

Name	Structure	Mw	Ions detected	MS/MS CID transitions (quantification ion in bold)	LOD (ng/µL)	LOQ (ng/µL)
Aldosterone		360	[M + H]^+^	**361** → **315,** 329, 343	0.17	0.39
Corticosterone		346	[M + H]^+^	**347** → **293**, 311, 329	0.039	0.064
Cortisol		362	[M + H]^+^	**363** → **327**, 345, 299	0.43	0.48
*β*-estrone		270	[M + H]^+^	**271** → **158,** 213, 353	0.0076	0.41

### Direct analysis of free steroids in the negative ion mode using PS-MS

**Figure 3:**
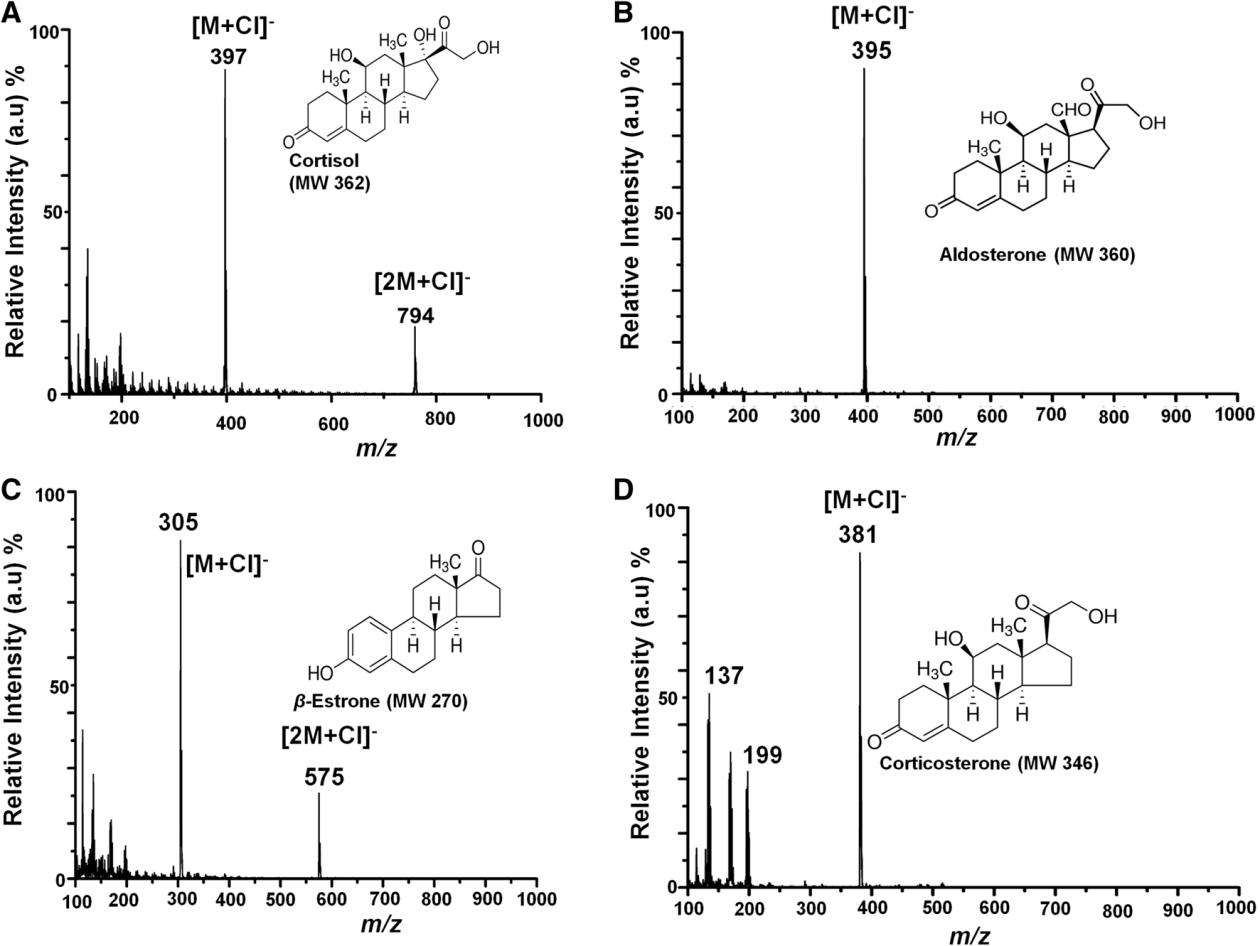
Negative ion mode paper spray mass spectrum of: (**A**) cortisol (Mw 362), (**B**) Aldosterone (Mw 360), (**C**) *β*-estrone (Mw 270) and (**D**) corticosterone (Mw 346) model steroid compounds, analyzed using a bench-top ion trap mass spectrometer with MeOH/H_2_O (1:1) doped with 0.05 mM HCl. Absolute amounts of analyte spotted onto a filter paper and ionized in open air by application of an electric potential (5 kV), 5 µL, viz 5 ng/µL.

Steroids in the negative ion mode were also investigated using MeOH/H_2_O (1:1, v/v) doped with formic acid or HCl. The standard model compounds were not detected using the standard PS solvent MeOH/H_2_O (1:1, v/v). Even the addition of a weak acid dopant (500 mM formic acid) did not favor any ionization for the model steroid compounds in the negative mode. However, upon the addition of a stronger acid dopant (0.05 mM of HCL) to the PS spray solvent, all of the steroid analytes analyzed could form stable ^35^Cl^-^ adducts, [M + ^35^Cl]^−^, with high intensity in the negative ion mode (Fig. 3A–D). Gas phase ion/molecule reactions can readily take place during the ionization process during paper spray analysis. It was found that the free steroids formed chloride adducts [M + Cl]^−^ in the negative ion mode when a diluted hydrochloric acid solution (0.05 mM) was doped in the paper spray solvent. The reactive paper spray experiment (Fig. 3) can, therefore, enhance the specificity of free steroid detection from complex sample mixtures. Moreover, the stability of the chloride adducts [M + Cl]^−^ affords improved sensitivity, perhaps due to the higher affinity of chloride ions compared to protons. This is also supported by the CID experiments of free steroid chloride adducts [M + Cl]^−^, which gave no fragments with the highest possible energy setting for collisions in our instrument (200 eV), demonstrating the stability of the chloride adducts, [M + Cl]^−^. As such, reliable identification can be achieved using exact mass measurement experiments of the parent ions from a high-resolution instrument^69^. The results obtained for the analysis of steroids in the negative ion mode are in agreement with those previously reported for the analysis of free and total cortisol in complex mixtures using LC-ESI with tandem mass spectrometry^66^.

### PS-MS analysis of steroid mixture

**Figure 4:**
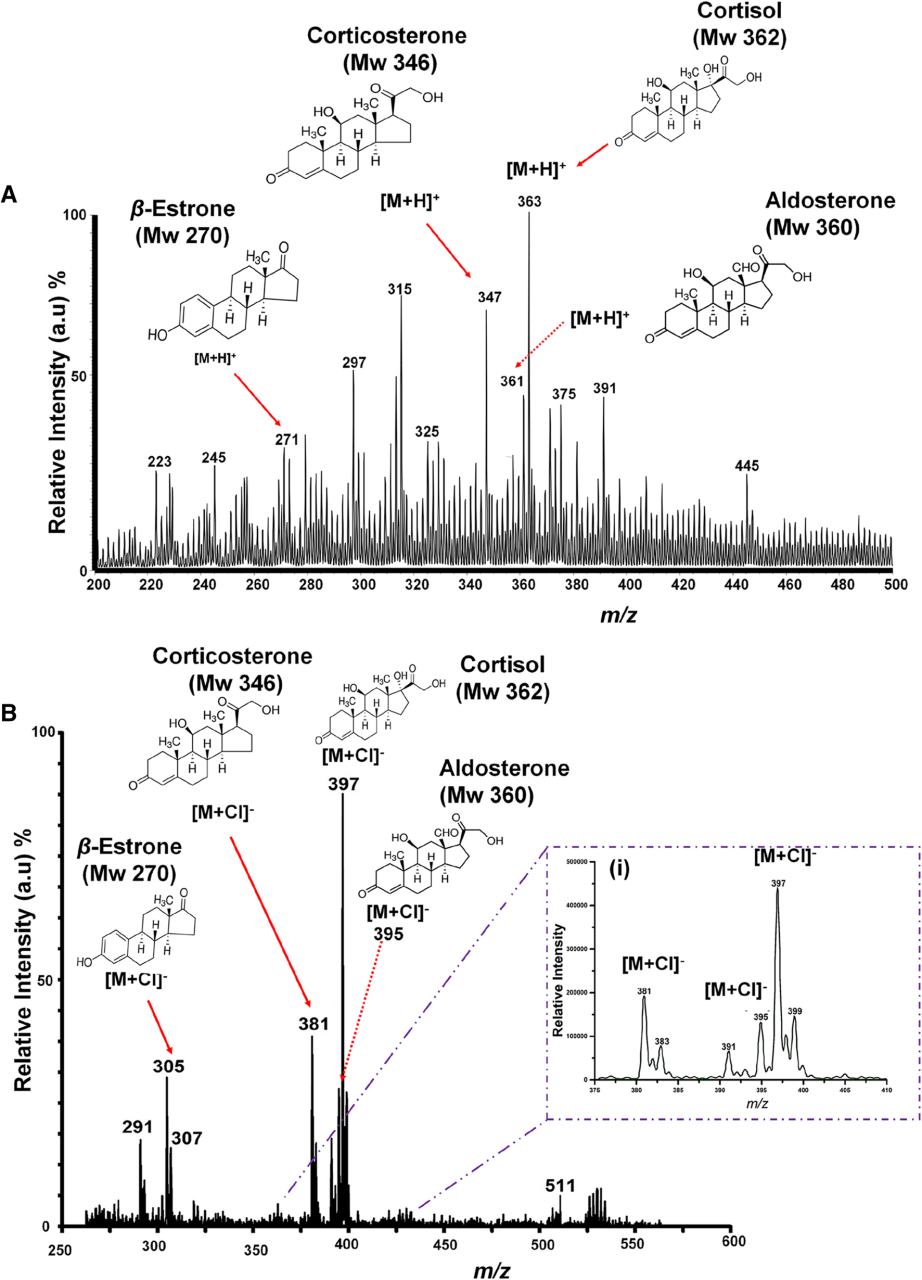
PS-MS analysis of the steroid model compounds (aldosterone (Mw 360), cortisol (Mw 362), *β*-estrone (Mw 270) and corticosterone (Mw 346)) in a mixture using a bench-top instrument. 5 µL, viz 10 µg/mL of analytes contained in the mixture were deposited onto the paper spray substrate. Ionization was performed in the open environment without any sample preparation by application of an electric potential of + 5 kV and − 5 kV in the positive and negative ion mode, respectively, using 20 µL MeOH/H_2_O (1:1, v/v) spray solvent doped with 0.05 mM of HCl as the PS-MS solvent: (**A**) relates to the positive mode, were model compounds in the mixture gave intact protonated [M + H]^+^ molecular peaks, and, (**B**) relates to the negative ion mode were Cl^−^ adducts are obtained. Insert (i) in (**B**), is a zoomed in portion of the spectrum illustrating the expected chorine isotopic distribution.

In this experiment a multicomponent stock solution of the model steroids (i.e. corticosterone (Mw 346), cortisol (Mw 362), aldosterone (Mw 360), *β*-estrone (Mw 270)) consisting of 10 µg/mL of each component in methanol solution was made up. The mixture was then analyzed using PS-MS operated in both positive and negative ion modes. A 5 μL aliquot of the matrix solution was spotted onto the paper substrate and analyzed using the commercial ion trap bench-top mass spectrometer. Figure 4A shows the mass spectra obtained from the analysis of the steroid artificial mixture using PS-MS in positive mode. In negative mode, the ability to form [M + Cl]^−^ adducts (Fig. 4B) with all the studied model compounds greatly simplifies the resulting mass spectra for mixture analysis without any prior separation. This approach can potentially be very useful for field applications, when coupled with miniaturized mass spectrometers.

### Quantification of free steroids in fish water samples

**Figure 5:**
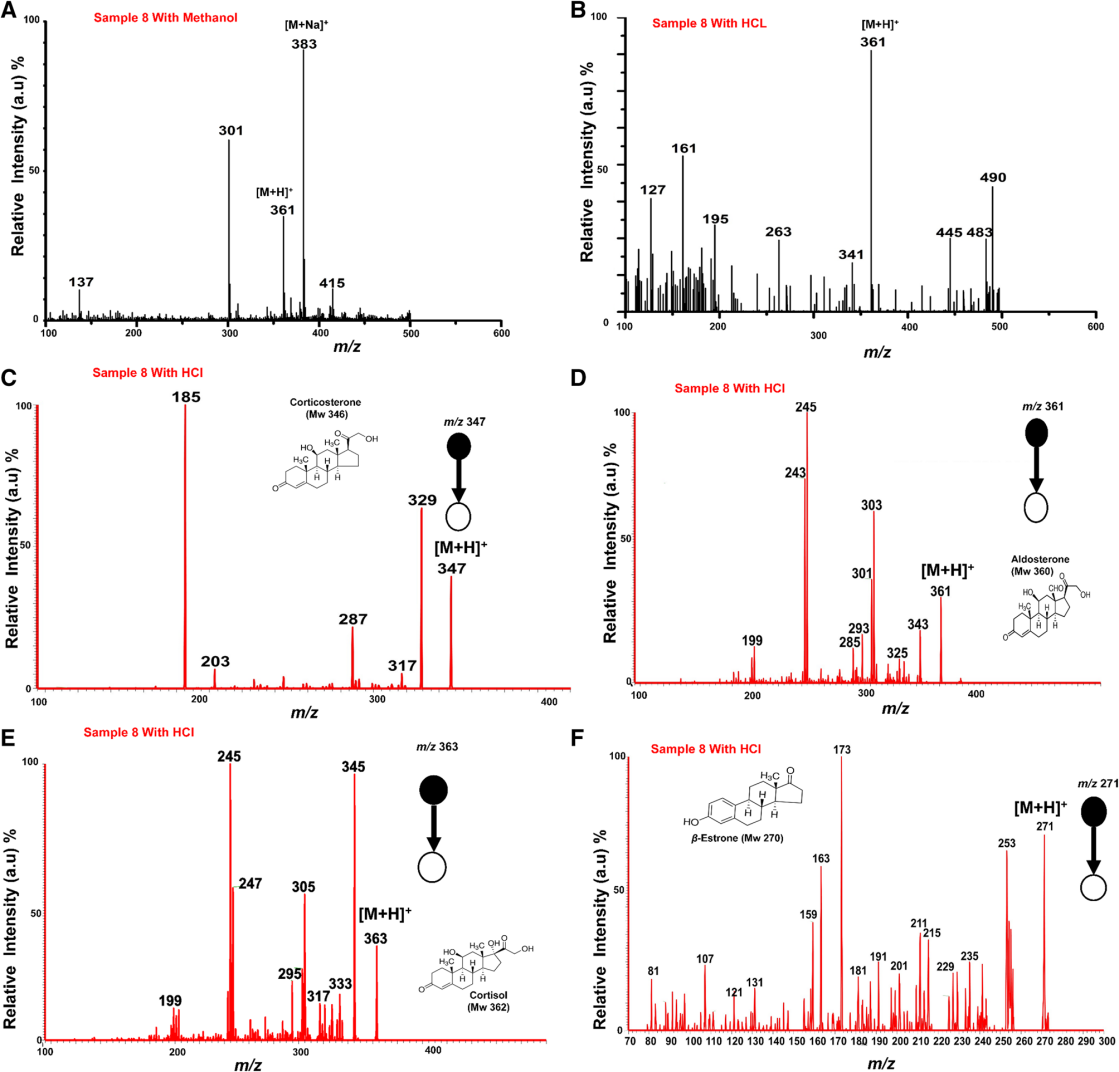
Positive ion mode paper spray mass spectra for the direct detection of free steroids in raw fish water samples. Approx. 5 μL volume of sample was deposited onto the paper substrate and ionized in the open environment by application of an electric potential of + 5 kV. Fish water sample 8 was analyzed using MeOH/H_2_O (1:1) as the spray solvent in (**A**). MeOH/H_2_O doped with 0.05 mM of HCl as the spray solvent in (**B**). (**C**)–(**F**) are the MS/MS CID mass spectra for the protonated molecular species, [M + H]^+^, of: (**C**) corticosterone at *m/z* 347, (**D**) aldosterone at *m/z* 361, (**E**) cortisol at *m/z* 363, and (**F**) *β*-estrone at *m/z* 271, all with HCl doped in the spray solvent.

The analysis of cortisol, aldosterone, corticosterone and *β*-estrone in real aquaculture water samples was investigated using PS-MS without any sample preparation. Thirteen (13) raw water samples were collected from fish tanks representing various conditions (not known to the authors), supplied by a fish farming company (who cannot be disclosed for commercial reasons), without any prior sample workup. A 5 μL volume from each raw sample was deposited onto individual paper substrates and analyzed using a commercial benchtop mass spectrometer in positive ion mode. Figure 5 shows a representative mass spectrum from one of the raw fish water samples (sample 8). When using the standard solvent system, a moderately intense ion at *m*/*z* 361 is observed, suspected to be a protonated aldosterone molecular ion, as well as an intense peak at *m*/*z* 383 suspected to be its corresponding sodium adduct, [M + Na]^+^ (Fig. 5A). In support of our suspicions as to the identity of these peaks, we then doped the spray solvent with 0.05 mM of HCl and a quite distinct mass spectrum was observed (Fig. 5B). The addition of a strong acid suppresses the sodiation ionization process favoring protonation of the steroid compounds in the mixture. Hence in Fig. 5B, only the protonated molecular ion species, [M + H]^+^, of aldosterone, at *m/z* 361, is distinguishable in the full MS mode; its identity was confirmed using MS/MS CID (Fig. 5D). The other steroid compounds (corticosterone, cortisol and *β*-estrone) were also observed in the raw sample and their identities confirmed by MS/MS CID experiments, as shown in Fig. 5 C, E, F (which all utilized PS solvent MeOH/H_2_O, doped with 0.05 mM of HCl). Expectedly the presence of steroids in the samples could not be confirmed in the same fish water samples when analyzed with the ‘normal PS-MS’, using PS solvent MeOH/H_2_O (1:1, v/v). Table 2 summarizes the results for the quantification of the free steroids in the raw fish water samples analyzed using PS-MS/MS. The mass spectra for samples 1–13 (except sample 8) are shown in the supplementary information in Figures S4–S15.

**Table 2:** Direct detection of free steroids in raw fish water samples analyzed using PS-MS.

Water sample	Absolute concentration of steroids ng/µL in fish water samples			
	Aldosterone	Corticosterone	Cortisol	*β*-Estrone
*S no. 1*	1.08 ± 0.11	0.60 ± 0.08	21.65^xx^ ± 0.46	6.74 ± 0.03
*S no. 2*	4.27 ± 0.09	1.22 ± 0.07	52.57^xx^ ± 1.24	3.66 ± 0.03
*S no. 3*	1.13 ± 0.11	0.66 ± 0.08	16.56^xx^ ± 0.34	3.81 ± 0.03
*S no. 4*	2.48 ± 0.10	1.00 ± 0.07	20.25^xx^ ± 0.43	9.30 ± 0.04
*S no. 5*	0.80 ± 0.11	0.62 ± 0.08	8.61 ± 0.17	1.76 ± 0.03
*S no. 6*	0.63 ± 0.11	0.26 ± 0.08	6.13 ± 0.13	4.09 ± 0.03
*S no. 7*	1.81 ± 0.10	0.67 ± 0.08	24.56^xx^ ± 0.54	2.93 ± 0.03
*S no. 8*	1.23 ± 0.11	1.36 ± 0.07	27.16^xx^ ± 0.60	4.05 ± 0.03
*S no. 9*	2.13 ± 0.10	0.81 ± 0.08	36.48^xx^ ± 0.84	4.31 ± 0.03
*S no. 10*	1.46 ± 0.10	0.52 ± 0.08	35.67^xx^ ± 0.81	3.52 ± 0.03
*S no. 11*	0.78 ± 0.11	0.31* ± 0.08	20.32^xx^ ± 0.43	4.48 ± 0.03
*S no. 12*	1.54 ± 0.10	0.79 ± 0.08	34.79^xx^ ± 0.79	4.55 ± 0.03
*S no. 13*	ND	0.18 ± 0.08	1.07 ± 0.14	10.65^xx^ ± 0.05

^xx^Analyte concentrations were extrapolated above the highest calibrator; *ND* not detected.

The analysis of free steroids in fish water using MeOH/H2O (1:1, v/v) as the PS solvent does not yield suitable results due to ion suppression. This is due to the salt concentration variations, which are likely to be encountered in most biological and environmental samples. Doping the standard PS solvent, MeOH/H2O (1:1, v/v), with a strong acid such as HCl enables protonation of free steroids in complex water samples to generate stable [M + H]^+^ species with improved sensitivity for target molecules present in complex mixtures. Although the inclusion of a fractional amount of a weak acid is common practice for MS techniques to aid protonation, the addition of a stronger acid, HCl, to the PS solvent for the suppression of sodiated adduct, [M + Na]^+^, formation was more effective. We attribute the high sensitivity of the protonated ion type to the occurrence of only one major fragment ion.

In this study, free steroid hormones (aldosterone, corticosterone, cortisol and *β*-estrone) in water were detected using PS-MS. The detected free steroid hormones can be released into the water via three main routes; the bile, the urine and the gills^70^. We detected the presence of free steroid hormones in most of the raw samples tested. A potential factor contributing to the higher hormone concentrations detected in the samples relates to the fact that these compounds accumulate in water systems, particularly when there are very large populations (i.e., high stock density) as is to be expected in commercial aquaculture.

The method provides a good basis for relative measure of free steroids in a non-invasive manner to monitor a fish stock holistically. For example, cortisol levels were found to be relatively high in some samples. It is well known that fish which encounter a stressful stimuli launch an endocrine stress response to release glucocorticoids (i.e. cortisol) into the water through the gills, urine, feces or mucus^8^. The cortisol levels detected in the studied samples (Table 2) show some agreement with those reported in previous studies using HPLC–MS^71–73^. Typical stressors can occur when water renewal is limited and stocking densities are high as well as during transportation and vaccination^73^. Whilst the reported LODs herein are higher than those from other analytical methods (e.g., LC/MS, RIA), the detection of steroids at the endogenous level in water has been achieved. The detection of hormones in water using a non-invasive method (i.e., without disturbing fish) such as PS-MS at higher concentrations can open up the possibility of on-site monitoring but in order to monitor the stock holistically, it would also require information on biomass, water flow rate and tank volume to calculate actual hormone release rates^74^. The initial study presented herein demonstrates the possibility of this approach for rapid *in aqua* analysis. However, further optimization and validation studies would be required to extend this method for similar applications where other factors may be prominent (e.g., environmental factors, water chemistry (turbidity, pH), etc.).

## Conclusion

The ability to detect and characterize free steroids in raw water samples collected from fish water tanks has been demonstrated using PS-MS/MS in the positive mode. Negative ion mode operation also shows promise, however the stability of the chloride adduct prevented collisionally activated dissociation with the instrument used. Further studies could address this limitation and/or investigate the possibility of steroid analysis using paper spray in the negative ion mode using exact mass measurement with a high-resolution mass spectrometer. In positive mode, the detection limits obtained suggest that this approach is suitable for direct and rapid detection of free steroids in raw water, although, as might be expected, it registers higher detection limits than those that can be obtained from GC- and LC–MS analytical methods^24,75^. With the capability of PS-MS to perform direct analyses on unmodified samples in a simple manner, the method demonstrated in this study shows great promise for non-invasive *in aqua* monitoring of fish stocks holistically in aquaculture, with minimal disruption.

The method provides a basis for the relative measure of free steroids in a non-invasive manner without any intervention other than collection of a water sample. The manual approach used herein allows a result to be obtained within ~ 1 min from sampling. A key advantage is being able to monitor the holistic profile of steroid hormones secreted in to a body of water. However, a potential drawback of the free steroids measurement in water is that it effectively integrates the free steroid release of all members of a population. As such, larger inter-individual differences in, for example, plasma concentrations of individuals may make a greater contribution to the overall free steroid concentration as determined from the collective. Nevertheless, the simplicity of paper spray ionization and the ability to analyze raw fish water samples without sample preparation further enhances the potential for coupling to a portable or miniaturized mass spectrometer for on-site analysis. Such a system in operation could be of great benefit for the aquaculture industry (e.g., for routine health monitoring and quality control).

## Experimental methods

### Chemicals and standards

The chromatography paper used as sample substrate was Whatman Grade I cellulose (Whatman International Ltd, Maidstone, UK). The PS organic solvents used include: methanol (HPLC grade), formic acid (reagent grade ≥ 95%), and hydrochloric acid (0.1 mol/L (0.1 N), pH = 1.2 (H_2_O, 20 °C)). All the standard model compounds (i.e. cortisol, corticosterone, and aldosterone, *β*-estrone, cortisol-1α,2α-d_2_, (HPLC grade, ≥ 95%)) were purchased from Sigma-Aldrich (UK). Thirteen (13) raw water samples were received from a commercial aquaculture company and used without preparation.

### Sample preparation

Pure steroid model compounds (i.e. cortisol, corticosterone, and aldosterone, *β*-estrone, cortisol-1α,2α-d_2_ (HPLC (grade, ≥ 95%)) were diluted to a target concentration in methanol (HPLC grade). The raw aquaculture water samples were analyzed as supplied without any sample preparation or purification using PS-MS. 5 µL of the sample was pipetted onto a cut paper triangle and analyzed directly.

### PS-MS instrumentation

**Figure 6:**
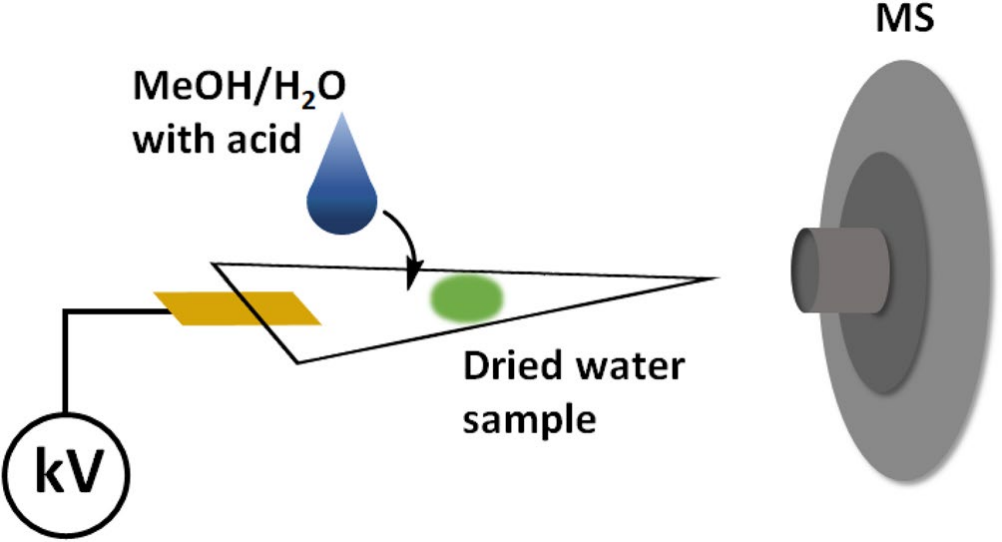
Schematic of the PS-MS experimental setup for the direct analysis of free steroids in water.

All steroid analysis was performed using a Thermo Scientific Velos Pro LTQ linear ion trap mass spectrometer (San Jose, CA, U.S.A.) tuned to the precursor ion of interest. The capillary temperature, capillary voltage, tube lens voltage were set to; 150 °C, 15 V, and 60% Slens voltage, respectively, in both positive and negative ion mode. Whatman grade 1 chromatography filter paper was used as the PS substrate. A DC voltage of ± 5.0 kV was applied on the PS substrate in all experiments. Figure 6 shows a sketch of the PS-MS experimental setup used in the analysis of free steroids in water.

A 5 µL aliquot of each sample was deposited on a filter paper surface followed by 20 µL of 1:1 MeOH/H_2_O (v/v) PS solvent, doped with either 500 mM formic acid or 0.05 mM HCl and analyzed directly without any sample preparation. Tandem mass spectrometry (MS/MS) was used for the structural elucidation, analyte identification and quantification. MS/MS was performed on the molecular ions of interest by collision-induced dissociation (CID) using an isolation window of 0.1–1.5 Th (mass/charge units) and normalized collision energy of 30–50 (manufacturers unit). Cortisol-D2 internal standard was spiked to a total of 5 ng/µL into each test sample and calibrator prior to spotting onto the paper surface. All quantification was performed by comparing the average intensity of the analyte fragment divided by the average intensity of the cortisol-D2 fragment 365 → 269 (IS). The ion transitions used for quantification were 347 → 293, 363 → 327, 361 → 315, and 271 → 158 for corticosterone, hydrocortisone, aldosterone, and estrone, respectively.

## Supplementary information

Supplementary file 1 (PDF 1946 kb)
