# Supplementary material for: Analysis of non-conjugated steroids in water using paper spray mass spectrometry
Source: Sci Rep. 2020 Jul 1;10:10698. doi: 10.1038/s41598-020-67484-7 (PMC7329809; doi:10.1038/s41598-020-67484-7)
Supplement: Supplementary file 1 — (PDF 1946 kb) [file 41598_2020_67484_MOESM1_ESM.pdf]

Supplementary Information

Analysis of Non-Conjugated Steroids in Water using Paper Spray Mass Spectrometry

Fred. P. M. Jjunju[¥], Deidre E. Damon[ƾ], David Romero-Perez[¥], Iain S. Young[+], Ryan. J Ward[¥], Alan Marshall[¥], Simon Maher[¥]*, Abraham K. Badu-Tawiah[ƾ,]*

[¥]Department of Electrical Engineering and Electronics University of Liverpool, Brownlow Hill, L69 3GJ, UK

[ƾ] Department of Chemistry and Biochemistry, The Ohio State University, Columbus, OH, 43210, USA

[+]Institute of Integrative Biology, University of Liverpool, Brownlow Hill, L69 7ZB, UK

*To whom correspondence should be addressed:

1. Dr. Abraham. K Badu-Tawiah, Department of Chemistry and Biochemistry, The Ohio State University, Columbus, OH, 43210, USA. Telephone: +1(614)-292-4276. Fax: (765) 494-9421. Email: badu-tawiah.1@osu.edu
2. Dr. Simon Maher, Department of Electrical Engineering and Electronics, University of Liverpool, Brownlow Hill, Liverpool, L69 3GJ, UK. Telephone: +44(0) 151 794 9517. Email: S.Maher@liverpool.ac.uk.

Keywords: Steroids, Ambient ionization, Paper Spray Mass Spectrometry, Water Quality Analysis, Aquaculture

Supplementary information is summarized in the table below

Topic	Title of Topic	Page
Topic 1 (Figures S1-S2)	Direct analysis of free steroids in the positive ion mode using PS-MS	3-4
Figure S3	Calibration curves for the model steroid compounds	5
Topic 2 (Figures S4-S15)	Quantification of free steroids in fish water samples	6 - 10

1. Direct analysis of free steroids in the positive ion mode using PS-MS

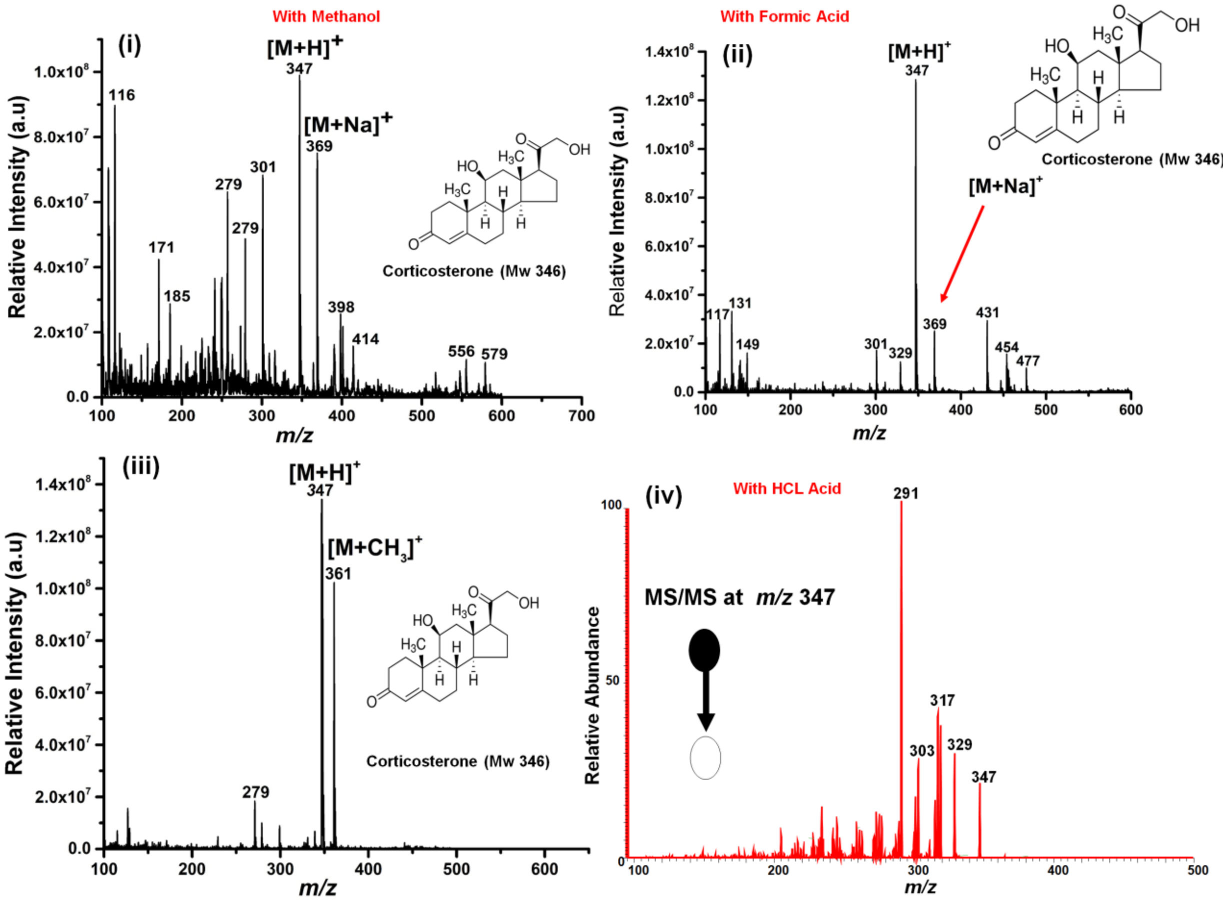

Figure S1. Figure 1. Positive ion mode paper spray mass spectra of corticosterone (MW 346) model compound analysed using a bench-top ion trap mass spectrometer; (i) with 10 µL 1:1 MeOH/H_2O, (ii) MeOH/H_2O (1:1) doped with 500 mM formic acid, (iii) MeOH/H_2O (1:1) doped with 0.05 mM HCl. In all cases absolute amounts of analyte were spotted onto a filter paper and ionized in the open air by application of an electric potential (+5 kV), 5 µL, viz 5 *ng*/µL. (iv) shows the MS/MS CID data for the selected protonated molecular ion, $[M+H]^+$, at *m/z* 347.

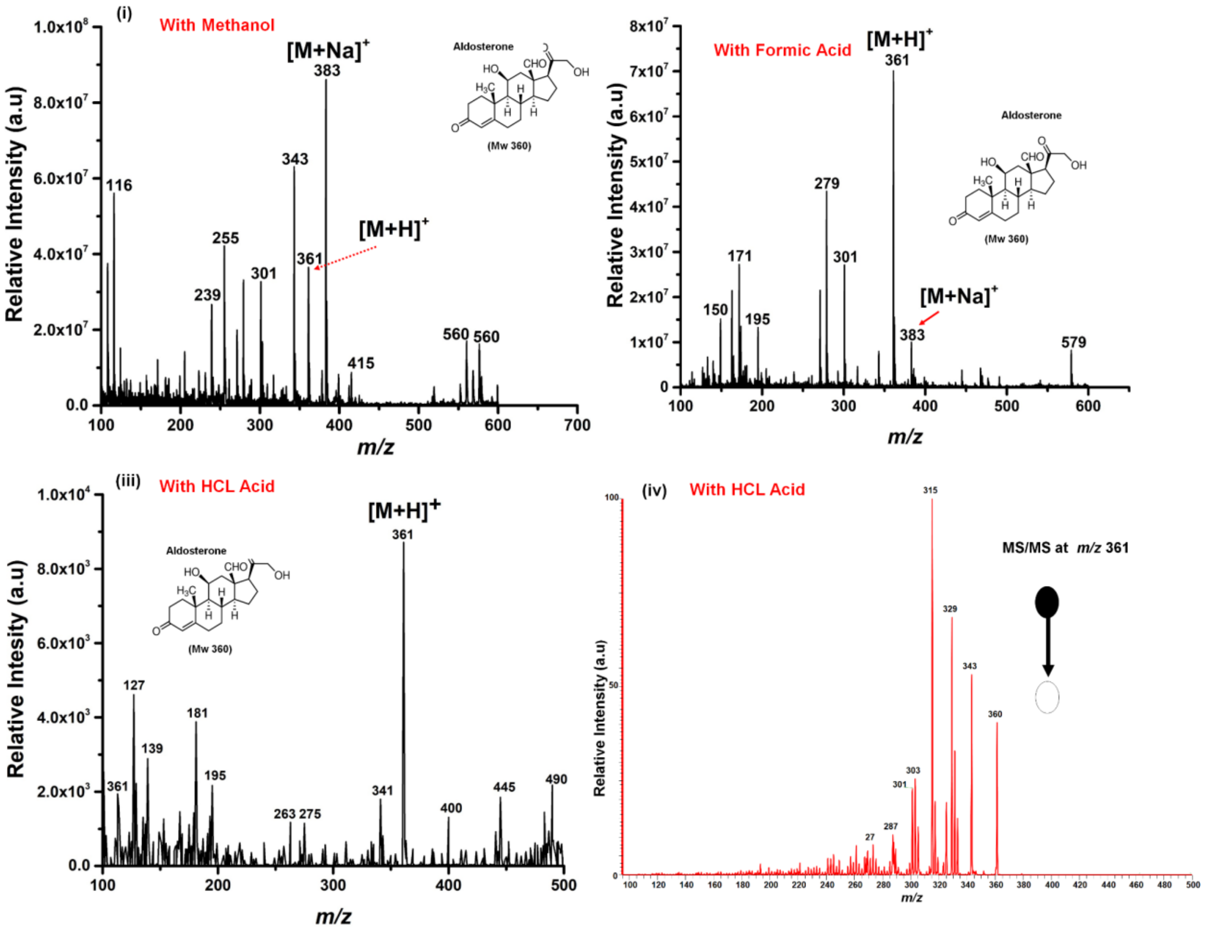

Figure S2. Positive ion mode paper spray mass spectra of aldosterone (MW 360) model compound analysed using a bench-top ion trap mass spectrometer; (i) with 10 µL 1:1 MeOH/H_2O, (ii) MeOH/H_2O (1:1) doped with 500 mM formic acid, (iii) MeOH/H_2O (1:1) doped with 0.05 mM HCl. In all cases absolute amounts of analyte were spotted onto a filter paper and ionized in the open air by application of an electric potential (+5 kV), 5 µL, viz 5 *ng*/µL. (iv) shows the MS/MS CID data for the selected protonated molecular ion, $[M+H]^+$, at *m/z* 361.

2. Limit of detection calibration curves for the free steroids in water using PS-MS

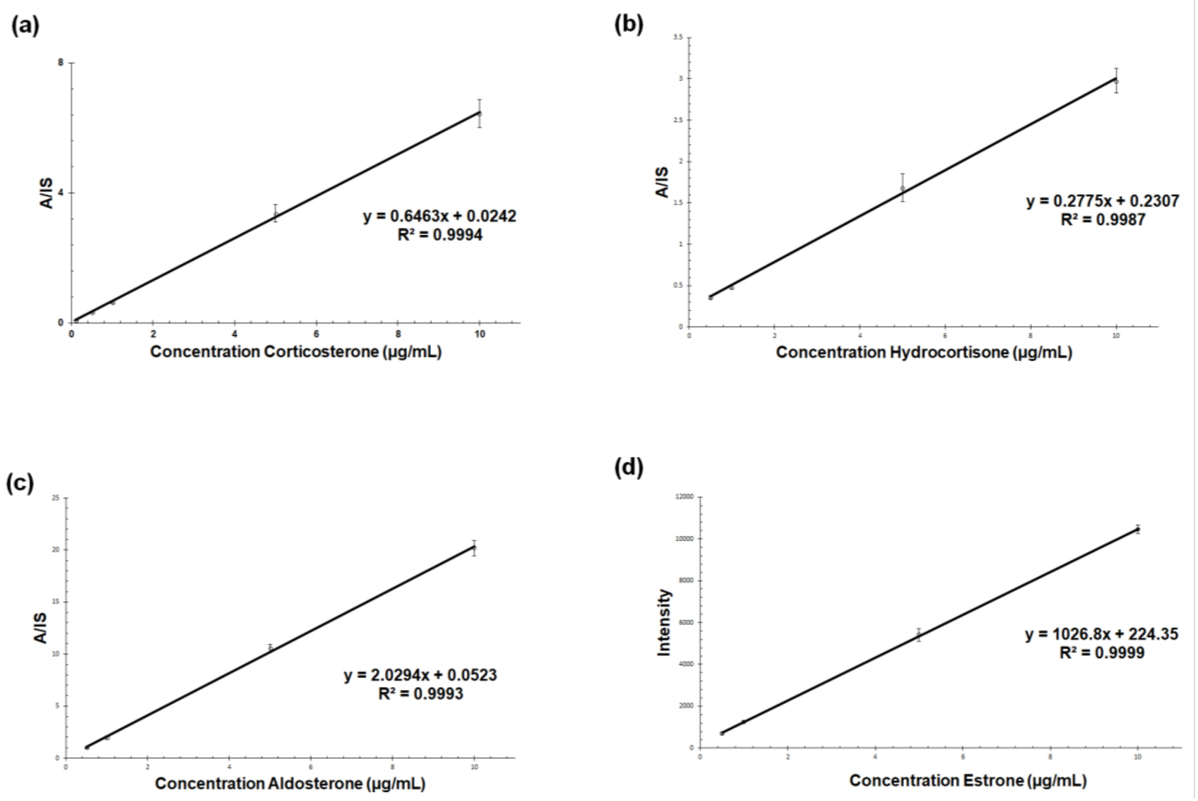

Figure S3. Calibration curves for the model standard steroid compounds in water analysed using MeOH/H_2O (1:1) doped with 0.05 mM HCl. Analyte was spotted onto a filter paper and ionized in open air by application of an electric potential (5 kV), 5 µL. (A) Corticosterone, (B) Cortisol, (C) Aldosterone and (D) *ß*-Estrone.

3. Quantification of free steroids in fish water samples

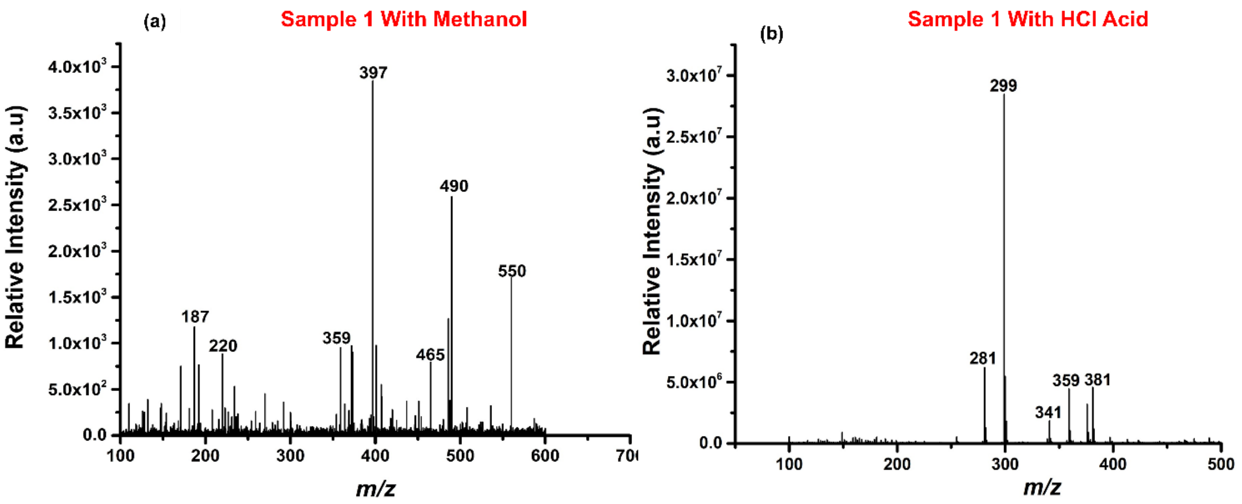

Figure S4. Positive ion mode paper spray mass spectra for the direct detection of free steroids in fish water samples. Approx. 5µL volume of sample was deposited onto the paper substrate and ionized in the open environment by application of an electric potential of +5 kV. Fish water sample 1 was analysed using MeOH/H_2O (1:1) (a) and (b) MeOH/H_2O (1:1), doped with 0.05 mM of HCl.

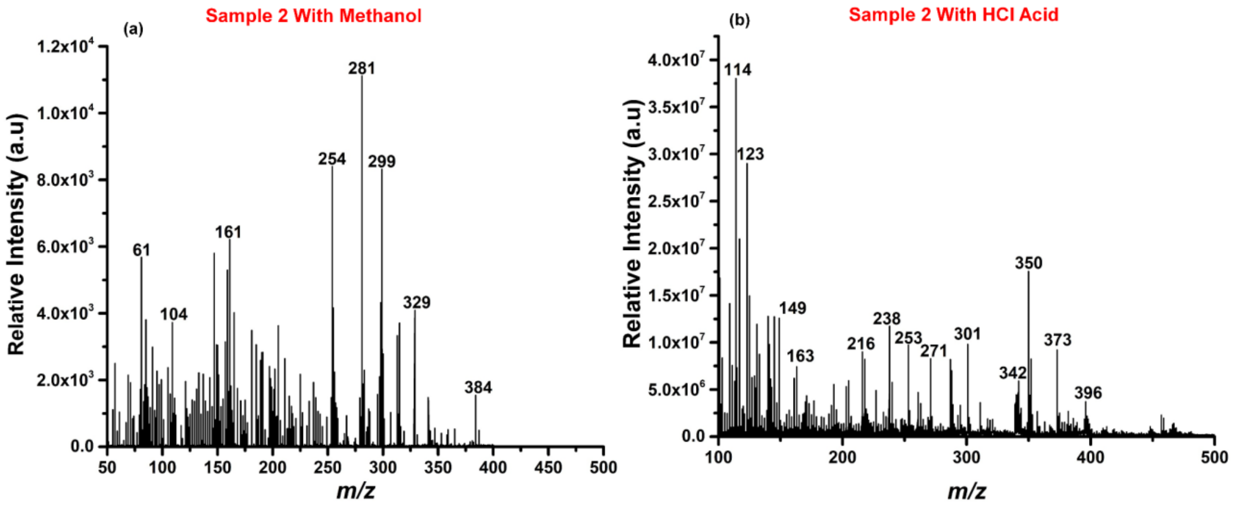

Figure S5. Positive ion mode paper spray mass spectra for the direct detection of free steroid in fish water samples. Approx. 5 µL volume of sample was deposited onto the paper substrate and ionized in the open environment by application of an electric potential of +5 kV. Fish water sample 2 was analysed using MeOH/H_2O (1:1) (a) and (b) MeOH/H_2O (1:1), doped with 0.05 mM of HCl.

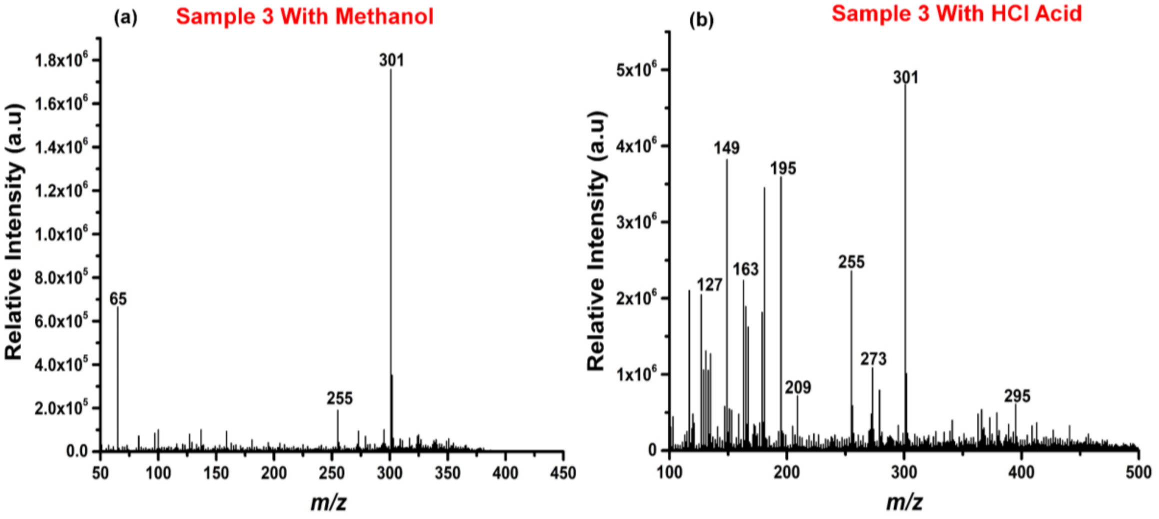

Figure S6. Positive ion mode paper spray mass spectra for the direct detection of free steroid in fish water samples. Approx. 5 µL volume of sample was deposited onto the paper substrate and ionized in the open environment by application of an electric potential of +5 kV. Fish water sample 3 was analysed using MeOH/H_2O (1:1) (a) and (b) MeOH/H_2O (1:1), doped with 0.05 mM of HCl.

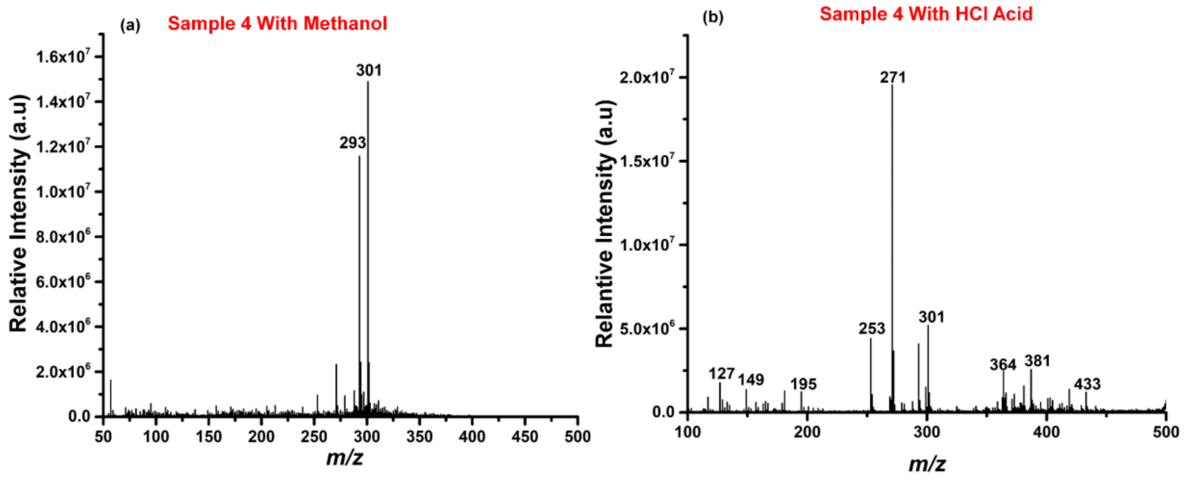

Figure S7. Positive ion mode paper spray mass spectra for the direct detection of free steroid in fish water samples. Approx. 5 µL volume of sample was deposited onto the paper substrate and ionized in the open environment by application of an electric potential of +5 kV. Fish water sample 4 was analysed using MeOH/H_2O (1:1) (a) and (b) MeOH/H_2O (1:1), doped with 0.05 mM of HCl.

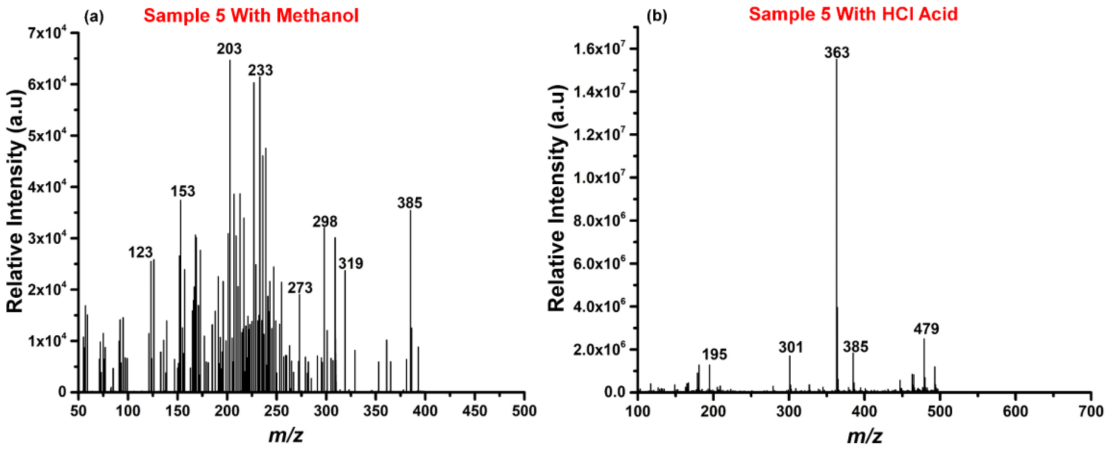

Figure S8. Positive ion mode paper spray mass spectra for the direct detection of free steroid in fish water samples. Approx. 5 µL volume of sample was deposited onto the paper substrate and ionized in the open environment by application of an electric potential of +5 kV. Fish water sample 5 was analysed using MeOH/H_2O (1:1) (a) and (b) MeOH/H_2O (1:1), doped with 0.05 mM of HCl.

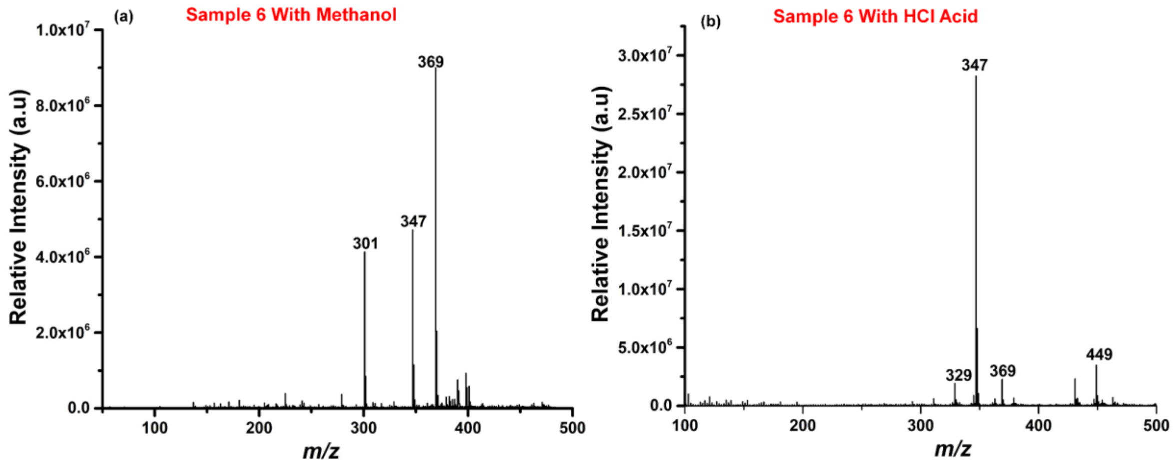

Figure S9. Positive ion mode paper spray mass spectra for the direct detection of free steroid in fish water samples. Approx. 5 µL volume of sample was deposited onto the paper substrate and ionized in the open environment by application of an electric potential of +5 kV. Fish water sample 6 was analysed using MeOH/H_2O (1:1) (a) and (b) MeOH/H_2O (1:1), doped with 0.05 mM of HCl.

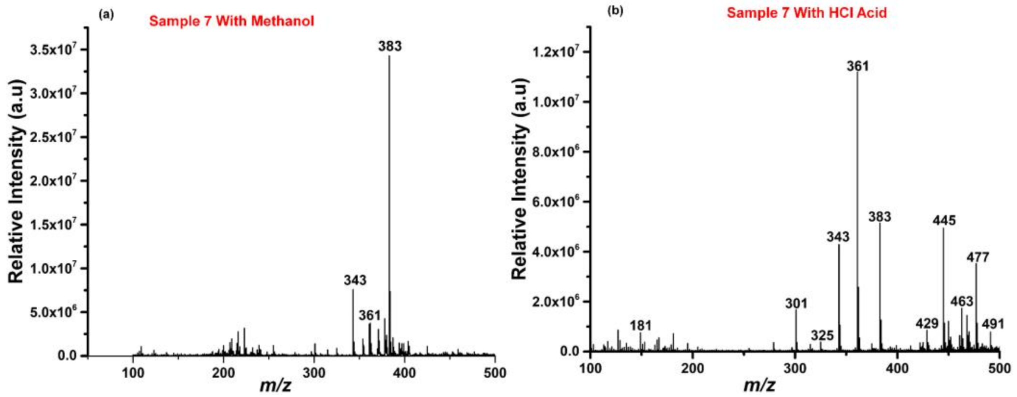

Figure S10. Positive ion mode paper spray mass spectra for the direct detection of free steroid in fish water samples. Approx. 5 µL volume of sample was deposited onto the paper substrate and ionized in the open environment by application of an electric potential of +5 kV. Fish water sample 7 was analysed using MeOH/H_2O (1:1) (a) and (b) MeOH/H_2O (1:1), doped with 0.05 mM of HCl.

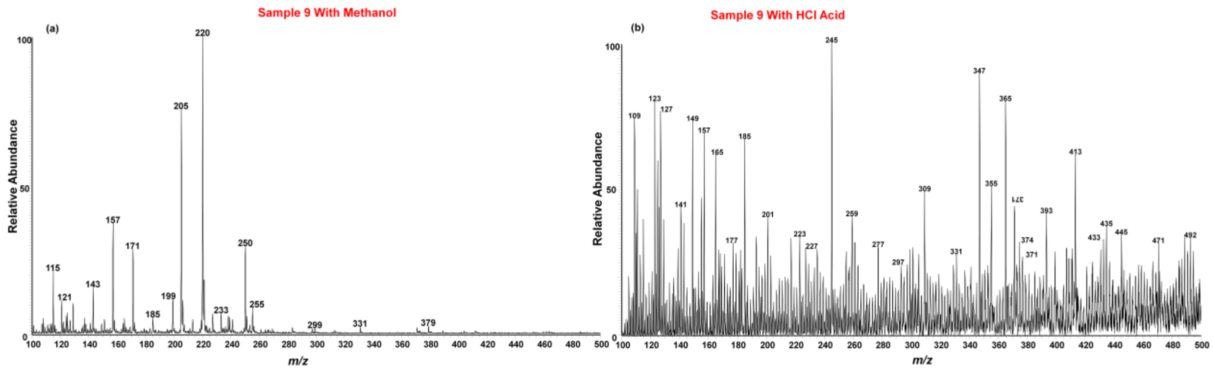

Figure S11. Positive ion mode paper spray mass spectra for the direct detection of free steroid in fish water samples. Approx. 5 µL volume of sample was deposited onto the paper substrate and ionized in the open environment by application of an electric potential of +5 kV. Fish water sample 9 was analysed using MeOH/H_2O (1:1) (a) and (b) MeOH/H_2O (1:1), doped with 0.05 mM of HCl.

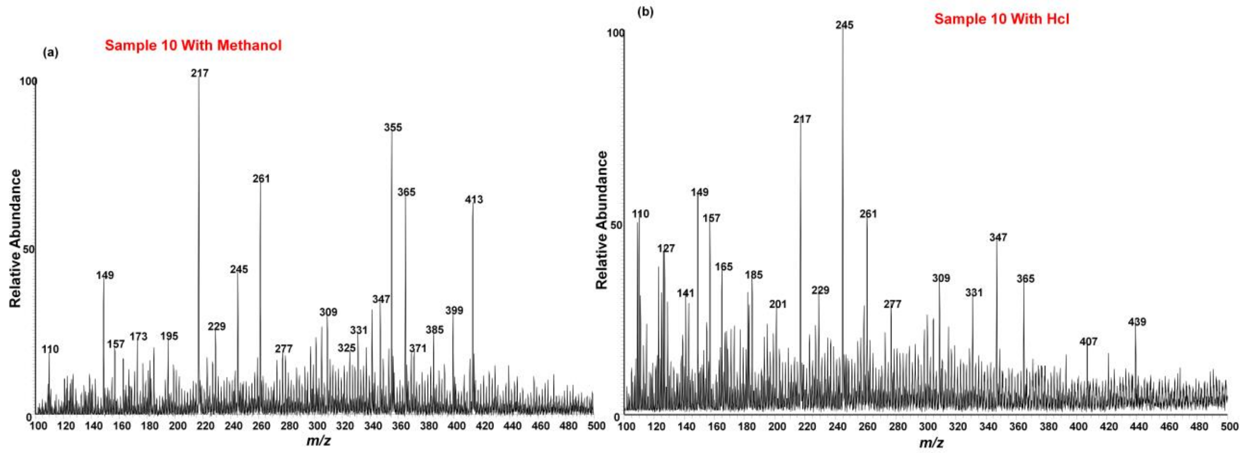

Figure S12. Positive ion mode paper spray mass spectra for the direct detection of free steroid in fish water samples. Approx. 5 µL volume of sample was deposited onto the paper substrate and ionized in the open environment by application of an electric potential of +5 kV. Fish water sample 10 was analysed using MeOH/H_2O (1:1) (a) and (b) MeOH/H_2O (1:1), doped with 0.05 mM of HCl.

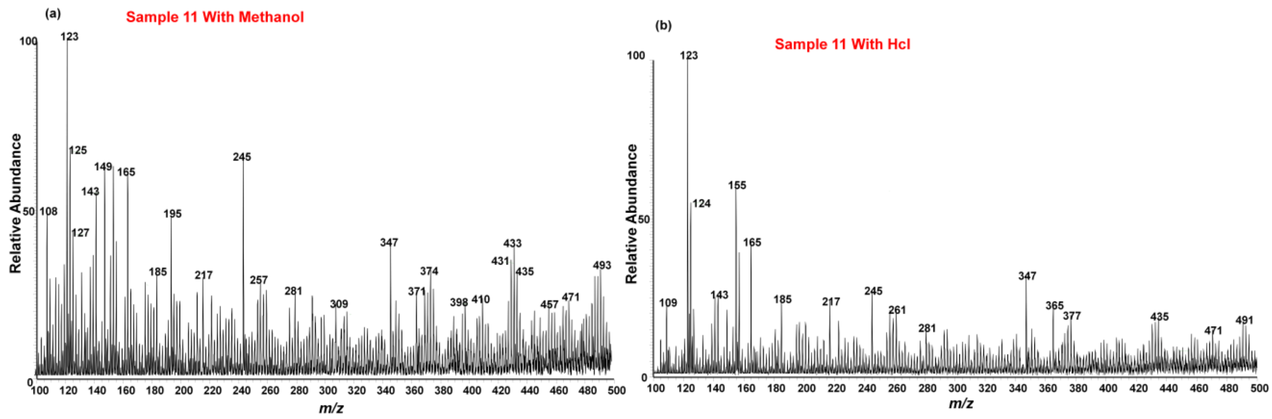

Figure S13. Positive ion mode paper spray mass spectra for the direct detection of free steroid in fish water samples. Approx. 5 µL volume of sample was deposited onto the paper substrate and ionized in the open environment by application of an electric potential of +5 kV. Fish water sample 11 was analysed using MeOH/H_2O (1:1) (a) and (b) MeOH/H_2O (1:1), doped with 0.05 mM of HCl.

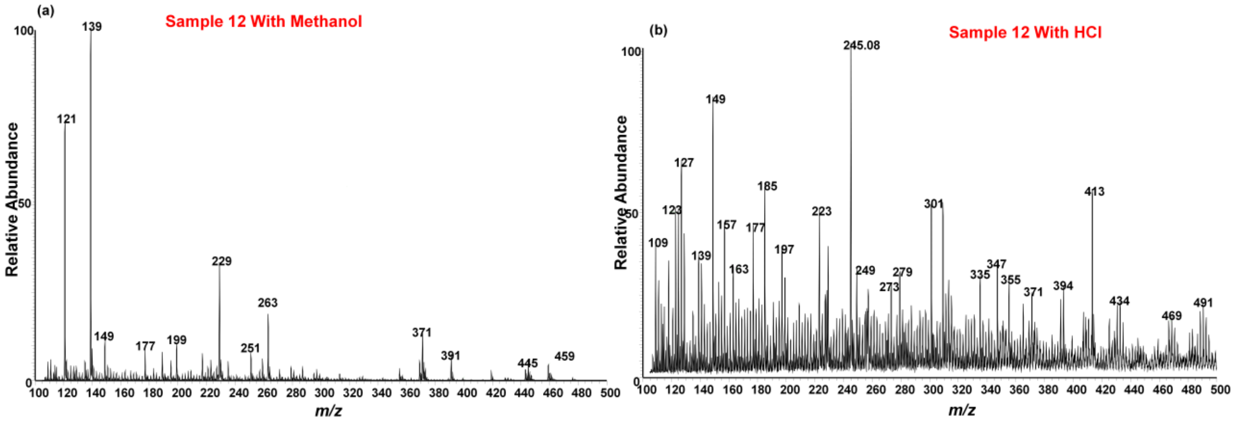

Figure S14. Positive ion mode paper spray mass spectra for the direct detection of free steroid in fish water samples. Approx. 5 µL volume of sample was deposited onto the paper substrate and ionized in the open environment by application of an electric potential of +5 kV. Fish water sample 12 was analysed using MeOH/H_2O (1:1) (a) and (b) MeOH/H_2O (1:1), doped with 0.05 mM of HCl.

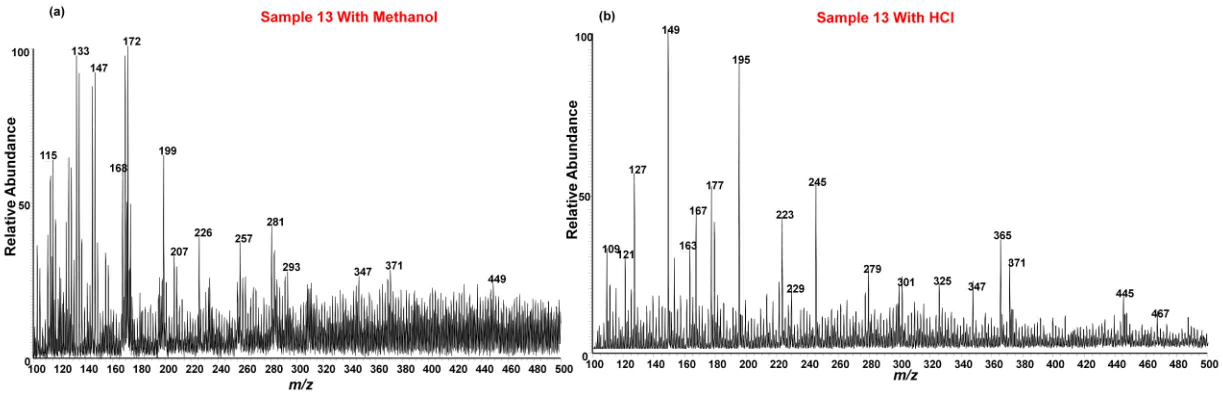

Figure S15. Positive ion mode paper spray mass spectra for the direct detection of free steroid in fish water samples. Approx. 5 µL volume of sample was deposited onto the paper substrate and ionized in the open environment by application of an electric potential of +5 kV. Fish water sample 13 was analysed using MeOH/H_2O (1:1) (a) and (b) MeOH/H_2O (1:1), doped with 0.05 mM of HCl.
